# Automated identification of uncertain cases in deep learning-based classification of dopamine transporter SPECT to improve clinical utility and acceptance

**DOI:** 10.1007/s00259-023-06566-w

**Published:** 2023-12-22

**Authors:** Thomas Budenkotte, Ivayla Apostolova, Roland Opfer, Julia Krüger, Susanne Klutmann, Ralph Buchert

**Affiliations:** 1 Department of Diagnostic and Interventional Radiology and Nuclear Medicine, University Medical Center Hamburg-Eppendorf, Martinistr. 52, 20246 Hamburg, Germany; 2 Jung Diagnostics GmbH, Hamburg, Germany

**Keywords:** Deep learning, Convolutional neural network, Uncertainty, FP-CIT, Dopamine transporter, SPECT

## Abstract

**Purpose:**

Deep convolutional neural networks (CNN) are promising for automatic classification of dopamine transporter (DAT)-SPECT images. Reporting the certainty of CNN-based decisions is highly desired to flag cases that might be misclassified and, therefore, require particularly careful inspection by the user. The aim of the current study was to design and validate a CNN-based system for the identification of uncertain cases.

**Methods:**

A network ensemble (NE) combining five CNNs was trained for binary classification of [^123^I]FP-CIT DAT-SPECT images as “normal” or “neurodegeneration-typical reduction” with high accuracy (NE for classification, NEfC). An uncertainty detection module (UDM) was obtained by combining two additional NE, one trained for detection of “reduced” DAT-SPECT with high sensitivity, the other with high specificity. A case was considered “uncertain” if the “high sensitivity” NE and the “high specificity” NE disagreed. An internal “development” dataset of 1740 clinical DAT-SPECT images was used for training (*n* = 1250) and testing (*n* = 490). Two independent datasets with different image characteristics were used for testing only (*n* = 640, 645). Three established approaches for uncertainty detection were used for comparison (sigmoid, dropout, model averaging).

**Results:**

In the test data from the development dataset, the NEfC achieved 98.0% accuracy. 4.3% of all test cases were flagged as “uncertain” by the UDM: 2.5% of the correctly classified cases and 90% of the misclassified cases. NEfC accuracy among “certain” cases was 99.8%. The three comparison methods were less effective in labelling misclassified cases as “uncertain” (40–80%). These findings were confirmed in both additional test datasets.

**Conclusion:**

The UDM allows reliable identification of uncertain [^123^I]FP-CIT SPECT with high risk of misclassification. We recommend that automatic classification of [^123^I]FP-CIT SPECT images is combined with an UDM to improve clinical utility and acceptance. The proposed UDM method (“high sensitivity versus high specificity”) might be useful also for DAT imaging with other ligands and for other binary classification tasks.

**Supplementary Information:**

The online version contains supplementary material available at 10.1007/s00259-023-06566-w.

## Introduction

Deep convolutional neural networks (CNN) are promising for many medical imaging tasks including the automatic interpretation of dopamine transporter (DAT)-SPECT images in clinically uncertain parkinsonian syndromes (CUPS). Several groups have trained and tested CNN for the automatic classification of CUPS [1–19]. However, the acceptance of these CNN for routine clinical use is limited by the lack of transparency (“black box nature”) and by the lack of certainty estimates of the automatic classification. In a review on molecular imaging in parkinsonism, Verger and co-workers concluded that CNN-based analyses outperform conventional machine learning methods such as support vector machines in terms of diagnostic performance but lack transparency, since they do not allow easy extraction of the features used to classify images of individual patients [20]. Since then, lack of transparency of CNN has been successfully addressed by “explainable AI” techniques providing a human readable explanation of the automatic classification decision that allows users to check if the CNN ‘s decision is plausible [11, 15].

In contrast, the lack of certainty estimates has not yet been addressed sufficiently. This is despite the fact that reporting the certainty of CNN-based decisions is highly desired to flag cases that might be misclassified by the CNN and, therefore, require particularly careful inspection by the user. There are different sources of uncertainty of CNN-based decisions. Uncertainty related to limitations of the CNN itself might be overcome by improving the network’s architecture and/or by more extensive network training. However, there are also true borderline cases that cannot be classified with high certainty also by expert readers. In DAT-SPECT of CUPS, the proportion of visually inconclusive borderline cases has been estimated at 5–10% [21, 22]. Automatic binary classification of these cases by a CNN might pretend a certainty of the diagnosis that is not actually given.

The most straightforward attempt to identify uncertain cases in CNN-based classification is to compute the distance of the CNN’s sigmoid output, ranging from 0 (most likely normal) to 1 (most likely reduced), to the predefined decision threshold on the sigmoid output (e.g., 0.5). However, this approach is not recommended among practitioners, as it tends to overestimate the certainty of CNN-based classification [23–25].

Against this background, the aim of the current study was to propose and validate a CNN-based uncertainty detection module (UDM) to identify DAT-SPECT that might be misclassified by automatic CNN-based classification.

## Materials and methods

### Datasets

The study retrospectively included three different datasets with a total of 3025 DAT-SPECT images (Table 1).

**Table 1:** Datasets

	Development dataset		Internal test dataset	External test dataset
	Training	Testing		
Number of scans	1250	690	640	645
Age (y)	66.9 ± 11.7	66.3 ± 11.4	67.2 ± 11.4	61.2 ± 10.2
Females (%)	43.0	44.9	44.2	35.2%
Number “reduced”/ “normal” scans (% “reduced”)	608/642 (48.6)	229/261 (46.7)	327/313 (51.1)	438/207 (67.9)

The primary dataset (“development dataset”) comprised 1740 consecutive DAT-SPECT from clinical routine at our site as described previously [26]. In brief, DAT-SPECT with [^123^I]FP-CIT had been performed according to common procedures guidelines [27, 28] with different double-head cameras equipped with low-energy-high-resolution or fan-beam collimators. The projection data were reconstructed using the iterative ordered-subsets-expectation–maximization [29] with attenuation and simulation-based scatter correction as well as collimator-detector response modelling implemented in the Hybrid Recon-Neurology tool of the Hermes SMART workstation v1.6 (Hermes Medical Solutions, Stockholm, Sweden) [30–33]. All parameter settings were as recommended by Hermes [30] for the EANM / EANM Research Ltd (EARL) ENC-DAT project (European Normal Control Database of DaTSCAN) [34–38]. More precisely, ordered-subsets-expectation–maximization was performed with five iterations and 15/16 subsets for 120/128 views. For noise suppression, reconstructed images were postfiltered by convolution with a three-dimensional Gaussian kernel of 7 mm full-width-at-half-maximum.

The development dataset was used for both, training and testing. For this purpose, the dataset was randomly split into 1250 training cases and 490 test cases.

The gold standard label as either “normal” or neurodegeneration-typical reduction (“reduced”) of the striatal signal had been obtained by visual interpretation of the DAT-SPECT images by three independent readers [26]. Each reader had performed two reading sessions of all images with a wash-out period between both sessions. Cases with intra-reader discrepant interpretation between the two sessions were assessed a third time by the same reader to obtain an intra-reader consensus. The majority vote across the three intra-reader consensus reads was used as gold standard for training and testing.

Among the 490 test cases from the development dataset, discrepancy across the six visual reads (three readers * two sessions) had been observed in 38 cases (7.8%). Visual intrepretation was concordant across the six reads in the remaining 452 cases (92.2%).

The second dataset (“internal test dataset”) comprised 640 consecutive DAT-SPECT with [^123^I]FP-CIT from clinical routine at our site that had been acquired with a triple-head camera equipped with brain-specific multiple-pinhole collimators. Multiple-pinhole SPECT concurrently improves count sensitivity and spatial resolution compared to SPECT with parallel-hole and fan-beam collimators [39, 40]. The projection data were reconstructed with the Monte Carlo photon simulation engine and iterative one-step-late maximum-a-posteriori expectation–maximization implemented in the camera software (24 iterations, two subsets) [40, 41]. Neither attenuation nor scatter correction was applied. The internal test dataset was used for testing only, not for training. The gold standard label (“normal” or “reduced”) was obtained by visual interpretation by an experienced reader (about 20 years of experience in clinical DAT-SPECT reading, ≥ 3000 cases). All SPECT images were interpreted twice (with different randomization) by the same reader. The delay between the reading sessions was 14 days. Cases with discrepant interpretation between the two reading sessions were read a third time by the same reader to obtain an intra-reader consensus as gold standard label.

The third dataset (“external test dataset”) comprised 645 DAT-SPECT with [^123^I]FP-CIT from the Parkinson’s Progression Markers Initiative (PPMI) (www.ppmi-info.org/data) [42]. The dataset included 438 patients with Parkinson’s disease and 207 healthy controls as described previously [18]. Details of the PPMI DAT-SPECT protocol are given at http://www.ppmi-info.org/study-design/research-documents-and-sops/ [42]. Raw projection data had been transferred to the PPMI imaging core lab for central image reconstruction using an iterative (HOSEM) algorithm on a HERMES workstation. The external test dataset was used for testing only, not for training. The clinical diagnosis was used as gold standard label (Parkinson’s disease = “reduced”, healthy control = “normal”).

**Fig. 1:**
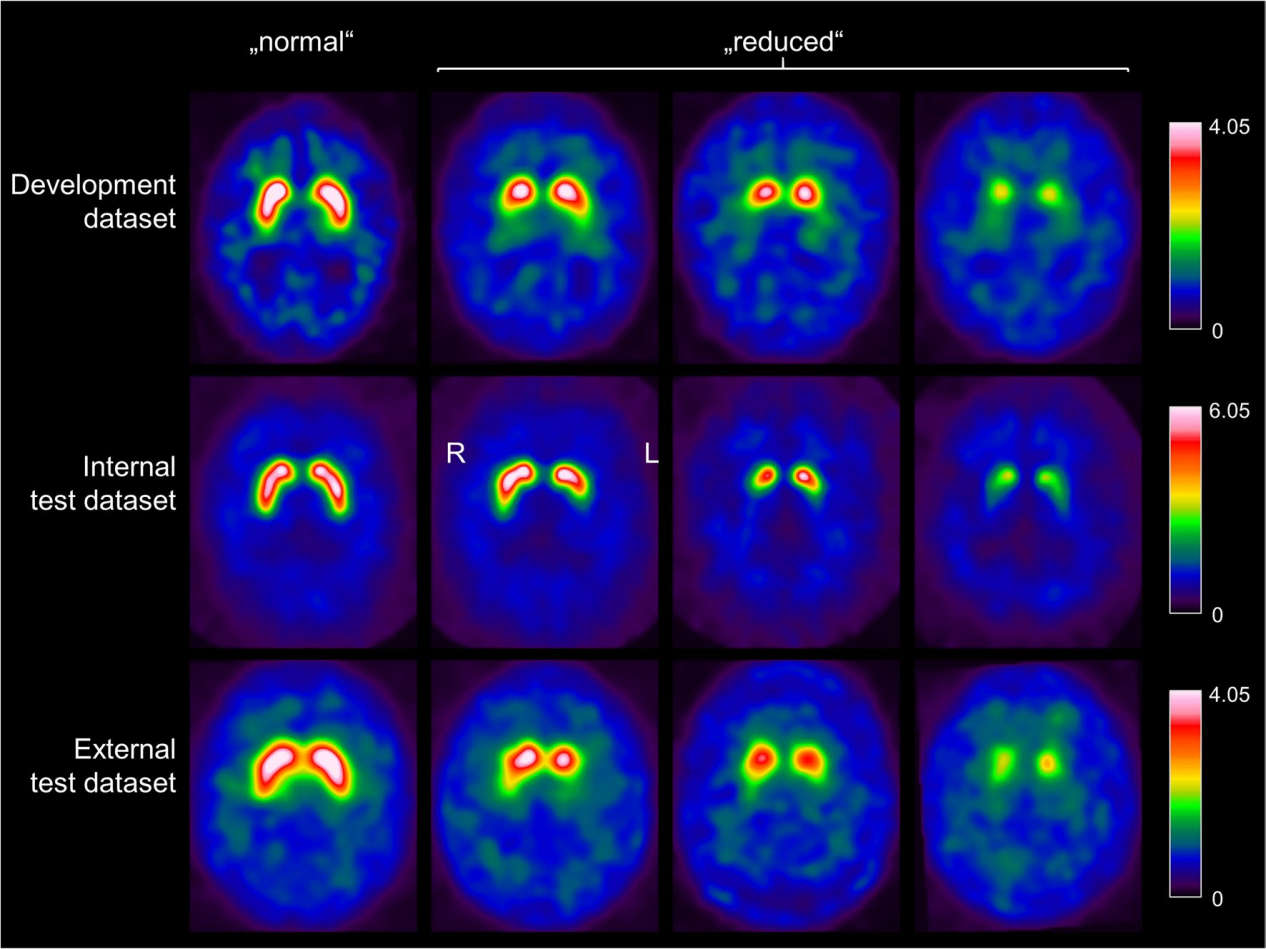
Two-dimensional slabs of 12 mm thickness representative of “normal” and “reduced” DAT-SPECT in the three datasets

Image characteristics were quite different between the three datasets (Fig. 1). Compared to the development dataset, the internal test dataset was characterized by better spatial resolution (resulting in higher striatum-to-background contrast) and less statistical noise. The external test dataset showed lower spatial resolution than the development dataset (lower striatum-to-background contrast). No attempts were made to harmonize the image characteristics across the datasets. In contrast, whereas the SPECT images in the development dataset were corrected for photon attenuation and scatter, the images in the internal test dataset were deliberately reconstructed without attenuation and scatter correction in order to further increase the between-datasets variability regarding the image characteristics. The rationale for this was to allow testing the UDM regarding its robustness with respect to between-site and between-camera variability of image characteristics.

### Image preprocessing

Individual DAT-SPECT images were stereotactically normalized (affine, no warping) to the anatomical space of the Montreal Neurological Institute using the Normalize tool of the Statistical Parametric Mapping software package (version SPM12) and a set of custom DAT-SPECT templates representative of normal and different levels of neurodegeneration-typical reduction of striatal uptake as target (moderate reduction more pronounced in the left hemisphere, moderate reduction more pronounced in the right hemisphere, strong bilateral reduction) [43]. Voxel size of the stereotactically normalized images was 2 × 2 × 2 mm^3^. Stereotactical normalization worked properly according to visual inspection in each of the 3025 DAT-SPECT included in this study.

Intensity normalization was achieved by voxelwise scaling to the individual 75^th^ percentile of the voxel intensity in a reference region comprising the whole brain without striata, thalamus, medial temporal lobe, brainstem, cerebellum, and ventricles [44]. The resulting images are distribution volume (DVR) images. A two-dimensional transversal DVR slab of 12 mm thickness and 91 × 109 pixels with 2 mm edge length was obtained by averaging six transversal slices through the striatum (Fig. 1) [45].

**Fig. 2:**
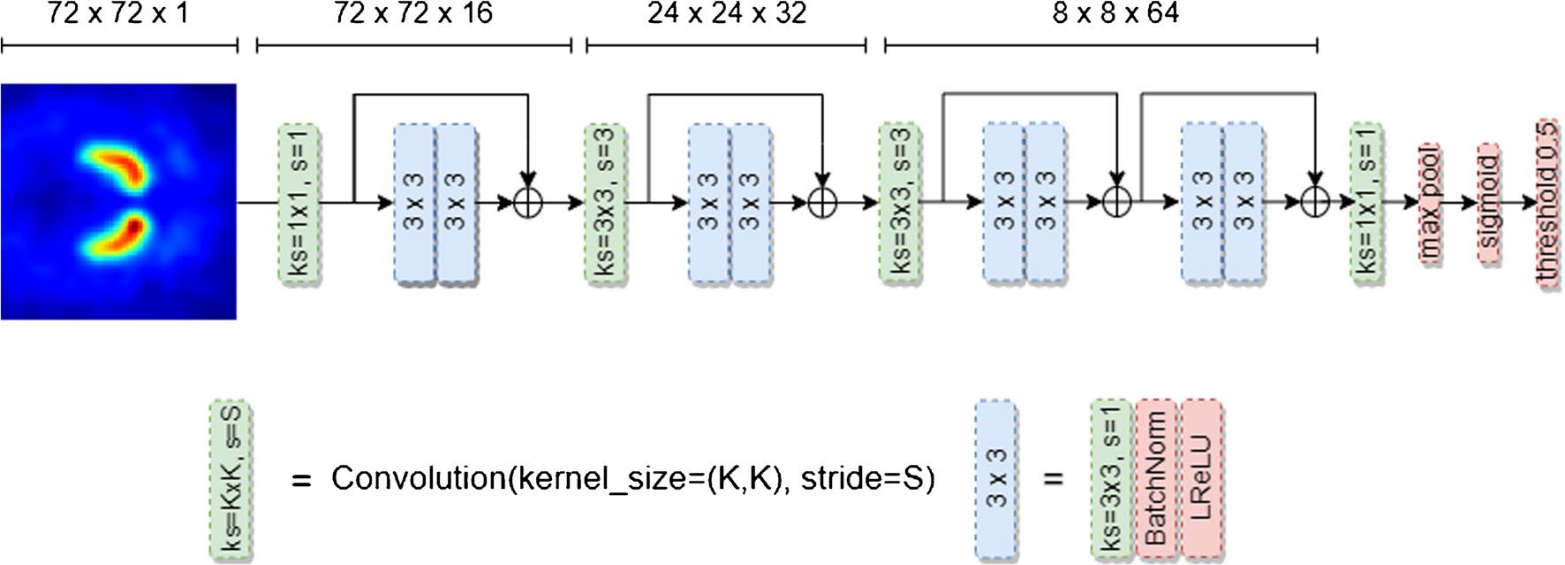
All CNN used in the current study had the same structure, comprising three stages with one, one, and two residual blocks (blue boxes) and 16, 32, and 64 filters per stage. Convolution with a 3 × 3 kernel and stride 3 for downsampling (green boxes) and batch normalization [46] were used at the beginning of the second and the third block. The final stage was followed by convolution with kernel size 1 × 1, a max reduction operator, and the sigmoid function to provide pseudo probabilities (ranging between 0 and 1) as output of the CNN. The CNN has 194,390 trainable parameters. This CNN structure had been selected in pilot experiments on reducing the CNN size without loss of performance in order to minimize inference costs and the risk of overfitting

A quadratic 72 × 72 DVR matrix centered at the striata was cropped from the DVR slab (Fig. 2). The DVR values were clipped to a maximum DVR of 6.5, and then z-tranformed using the global mean and the global standard deviation across all 72 × 72 pixels in all scans in the training sample from the development dataset (global mean and global standard deviation were used instead of z-transformation separately for each image in order to preserve the semi-quantitative DVR information in the images). Global mean and global standard deviation computed in the training dataset were also applied for z-transformation of the three test datasets. The resulting 72 × 72 matrices served as input to all CNN.

### CNN model for classification of DAT-SPECT

**Fig. 3:**
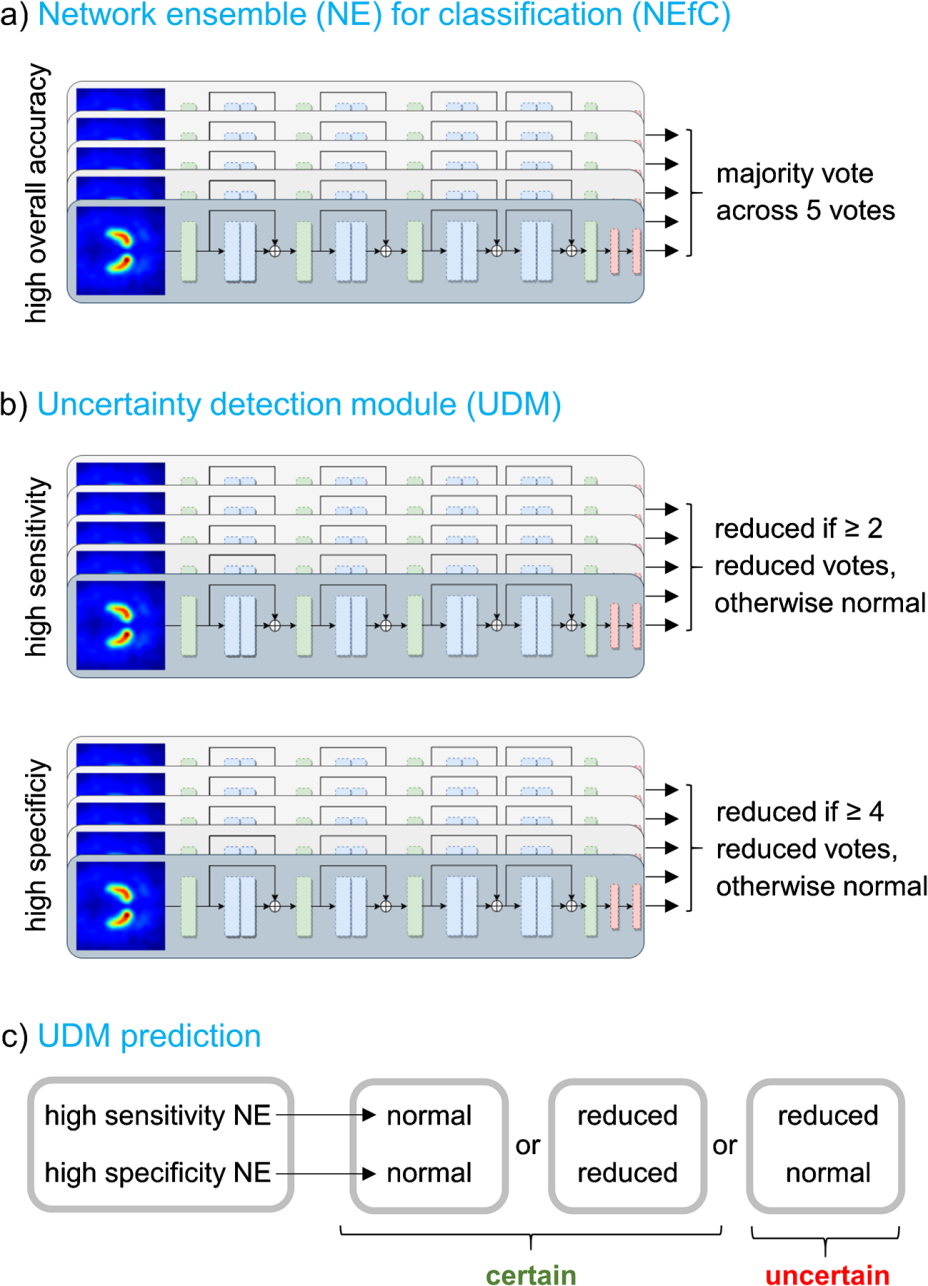
Workflow: The network ensemble (NE) for classification (NEfC, **a**) consists of five independent convolutional neural networks (CNN), separately trained for binary classification of DAT-SPECT with high overall accuracy. The majority vote across the five CNN of the NEfC is assumed to be the binary classification that most likely is correct. The uncertainty detection module (UDM, **b**) comprises two additional NE, one trained to detect reduced DAT-SPECT with high sensitivity, the other with high specificity. A DAT-SPECT is flagged as uncertein by the UDM, if the two NE of the UDM disagree in their binary categoriozation as normal or reduced (**c**). The same DAT-SPECT image serves as input to all CNN

A network ensemble (NE) combining five CNN was trained for classification of DAT-SPECT with high overall accuracy (NE for classification, NEfC). Each CNN in the ensemble had identical standard ResNet [47] structure (Fig. 2) but different random initialization by the HE initializer [48]. The cut-off 0.5 on the sigmoid output was used to generate a binary decision (“normal” or “reduced”), separately for each of the five CNN. The majority vote across the five CNN was used as binary decision of the NEfC (Fig. 3a).

The NEfC was trained (including tuning of hyper-parameters) from scratch using five-fold cross-validation in the 1250 training cases from the development dataset. Cross-entropy as loss function was optimized using a standard stochastic gradient descent optimizer with Nesterov momentum [49] of factor 0.9, a weight decay of magnitude 3e-5, and a linear warmup plus cosine decay learning rate schedule with maximum learning rate 3e-4. The batch size was set to 12. Each batch comprised the same number (n = 6) “normal” and “reduced” DAT-SPECT. During the training, data augmentation was performed on the fly according to the recommendations of the nnUNet framework [50]. Augmentation included spatial methods (rotation, grid scaling, flipping), and intensity-based methods (adding of Gaussian noise, Gaussian blurring, multiplicative intensity scaling, intensity clipping, and gamma transformations) [50]. The parameters used for data augmentation are given in Supplementary Table 1. The training of the NEfC took about 30 min on a single GPU in a standard deep learning workstation with an inference throughput of 30–100 images per second.

The NEfC yields a binary decision for each individual DAT-SPECT, it does not directly indicate the certainty of its decision.

### CNN-based uncertainty detection module

In order to identify DAT-SPECT in which the binary NEfC decision is uncertain, two additional NE each consisting of five CNN were trained for classification of DAT-SPECT using five-fold cross-validation in the 1250 training cases from the development dataset. One NE was intended to provide high sensitivity for the detection of reduced DAT-SPECT (“high sensitivity” NE, low rate of false negative cases), the other was intended to provide high specificity for this task (“high specificity” NE, low rate of false positive cases). If the two network ensembles *disagree* in their classification of a given DAT-SPECT image, this image is flagged as “uncertain”. The rationale behind this procedure is the following. If a DAT-SPECT image is categorized as “normal” by the high sensitivity NE, this is most likely correct, because this NE has been trained to avoid false negative classifications (at the expense of some higher risk of false positive ones). The high specificity NE most likely will also classify the case as “normal”, because it is trained to correctly identify as much as possible “normal” scans (at the expense of some higher risk of false negative classifications). The same argument holds vice versa for clearly reduced DAT-SPECT. However, borderline cases are expected to be categorized as “reduced” by the high sensitivity NE and as “normal” by the high specificity NE, because they have been trained to do so. Hence, the borderline cases can be identified by disagreement of the two NE.

For the training of the high sensitivity and the high specificity NE of the UDM, the following changes were implemented compared to the training of the NEfC [51, 52]: (i) weights were added in the cross-entropy loss function to penalize false negative decisions (for the “high sensitivity” NE) or to penalize false positive decisions (for the “high specificity” NE), (ii) 11-to-1 overrepresentation of “reduced” cases in the training batches (for the “high sensitivity” NE) or 11-to-1 overrepresentation of “normal” cases (for the”high specificity” NE), and (iii) alteration of the voting system: the binary decision of the “high sensitivity” (“high specificity”) NE was “reduced” (“normal”) if two or more of its CNN decided “reduced” (“normal”). The “high sensitivity” NE and the “high specificity” NE were trained independently.

The entire workflow is illustrated in Fig. 3. The NEfC trained for classification of DAT-SPECT with high overall accuracy (Fig. 3a) is used to obtain the binary decision that most likely is correct. In parallel, the independent UDM (Fig. 3b, c) is used to identify the cases in which this binary NEfC classification is “uncertain”.

For comparison, three established approaches for uncertainty detection were implemented: (i) using the sigmoid outputs of the five CNN in the fully trained NEfC (“sigmoid” method), (ii) applying dropout during training and inference (“dropout” method), and (iii) using a set of several NEfC fully trained with different random seeds for initialization (model “averaging” method). For the “sigmoid” method, the sigmoid output was averaged across the five CNN in the NEfC. A case was considered “uncertain” according to the “sigmoid” method if the mean sigmoid output was in the interval [0.5-t, 0.5 + t]. The “dropout” method applied dropout with probability 0.1 after each convolution of a residual block in each of the five CNN of the NEfC both, during training and during inference of the trained NEfC. For application to a given DAT-SPECT, the trained NEfC was applied seven times with different random dropout. This resulted in seven applications * 5 CNN = 35 sigmoid outputs. A case was considered “uncertain” according to the “dropout” method if the mean across the 35 sigmoid outputs was in the interval [0.5-t, 0.5 + t]. For the model “averaging” method, seven identical NEfC each consisting of five identical CNN were independently trained (starting with different random seeds) for classification of DAT-SPECT with high overall accuracy in the 1250 training cases from the development set. For a given DAT-SPECT, each of the seven NEfC was applied. This resulted in 7 NEfC * 5 CNN per ensemble = 35 sigmoid outputs. The case was considered “uncertain” according to the “averaging” method if the mean across the 35 sigmoid outputs was in the interval [0.5-t, 0.5 + t].

### Statistical analysis

The NEfC trained for classification of DAT-SPECT in the 1250 training cases from the development dataset was tested in the 490 test cases from the development dataset, in the independent internal test dataset and in the independent external test dataset. Overall accuracy, sensitivity and specificity were used as performance metrics.

The UDM and the three comparison methods were applied to the test cases from the development dataset, to the internal test dataset and to the external test dataset. In the test cases from the development dataset, the proportion of cases flagged as uncertain by the UDM was compared between the cases with fully concordant and the cases with discordant interpretation across the six visual reads that had been performed to generate the gold standard label. The rationale for this was that between-reads discrepancy is much more likely in borderline cases (that should be flagged as uncertain by the UDM) than in clear cases (that should not be flagged as uncertain). The threshold t required for uncertainty detection with each of the three comparison methods was fixed such that the proportion of “uncertain” cases was equal to the proportion of “uncertain” cases according to the UDM, separately for each of the comparison methods. The following metrics were used to characterize the performance of the UDM and the comparison methods: (i) the proportion of “uncertain” cases among the cases misclassified by the NEfC (as measure of the sensitivity to detect misclassified cases), (ii) the proportion of “uncertain” cases among cases correctly classified by the NEfC (as measure of the utility of the UDM), and (iii) the proportion of misclassified cases among the “certain” cases (as measure of the accuracy that can be achieved when restricting the automatic classification by the NEfC to “certain” cases).

## Results

Concerning automatic binary classification of DAT-SPECT, NEfC performance in the different test datasets is given in Table 2.

**Table 2:** Classification performance of the NEfC trained for high overall accuracy in the development dataset

Dataset	TP/TN/FP/FN	Overall accuracy	Sensitivity	Specificity
Training sample from the development dataset, five-fold cross-validation	597/630/12/11	0.982	0.982	0.981
Test sample from the development dataset	222/258/3/7	0.980	0.969	0.989
Internal test dataset	290/312/1/37	0.941	0.887	0.997
External test dataset	437/195/12/1	0.980	0.998	0.942

Classification performance of the “high sensitivity” and the “high specificity” NE of the UDM is given in Supplementary Table 2.

Concerning uncertainty detection, the UDM flagged 21 (4.3%) of all 490 cases in the test sample of the development dataset as uncertain. Among the 38 cases with discrepancy across the 6 visual reads, 15 (39.5%) were flagged as uncertain by the UDM. From the remaining 452 cases (all 6 visual reads concordant), 6 (1.3%) were flagged as uncertain.

The proportion of scans flagged as uncertain by the UDM in the internal test dataset and in the external test dataset was 3.9% and 6.5%, respectively. Thresholds t to achieve the same proportion of “uncertain” cases with the comparison methods are given in Supplementary Table 3.

**Fig. 4:**
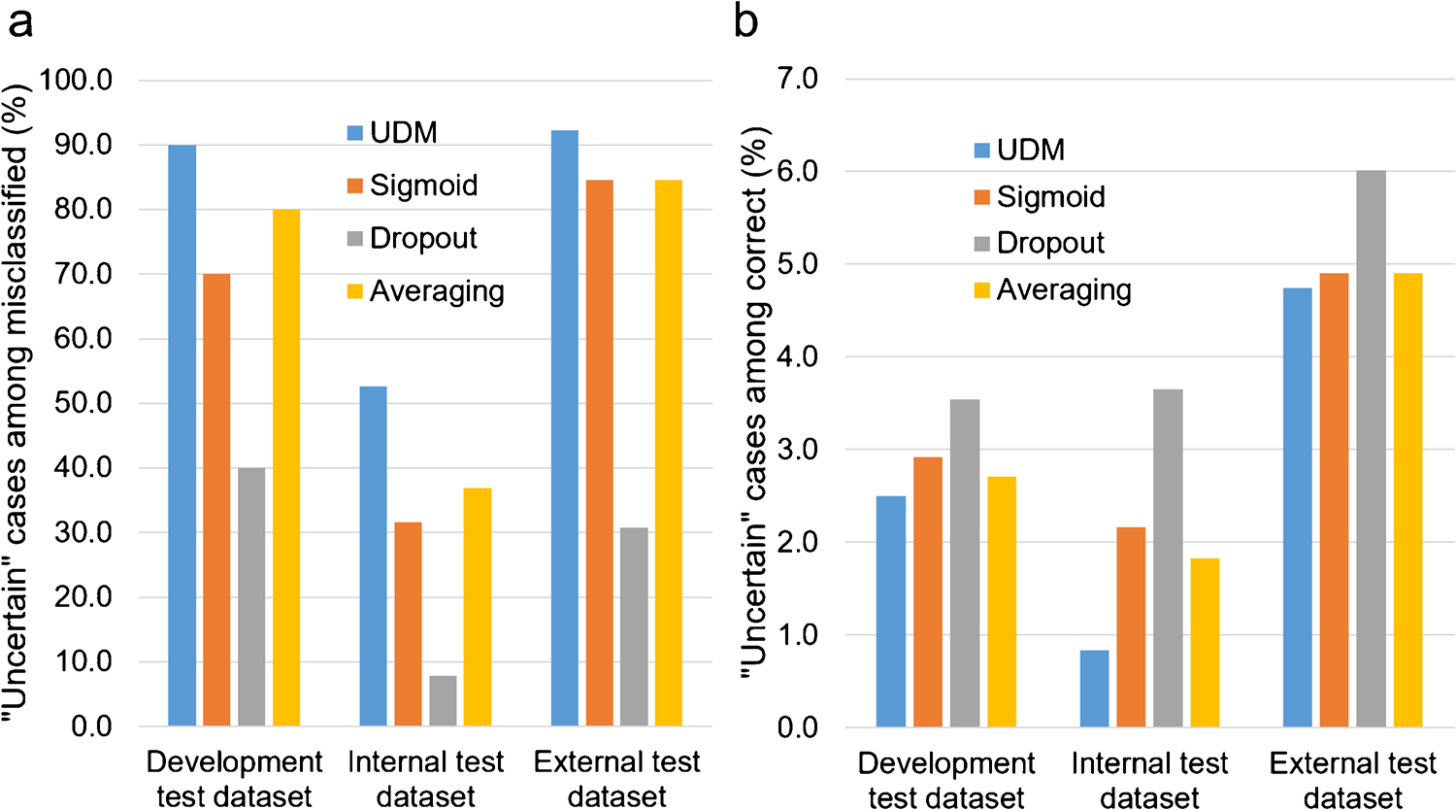
Uncertainty detection performance: proportion of “uncertain” cases among misclassified cases (by the NEfC) (**a**) and among correctly classified cases (**b**). For the three comparison methods (“sigmoid”, “dropout”, “model averaging”), the proportion of “uncertain” cases in the whole testset was fixed to be the same as for the UDM, separately for each testset (4.3%, 3.9%, and 6.5% for the test sample from the development dataset, the internal test dataset, and for the external test dataset, respectively)

The proportion of “uncertain” cases among misclassified cases and among correctly classified cases is shown in Fig. 4.

**Fig. 5:**
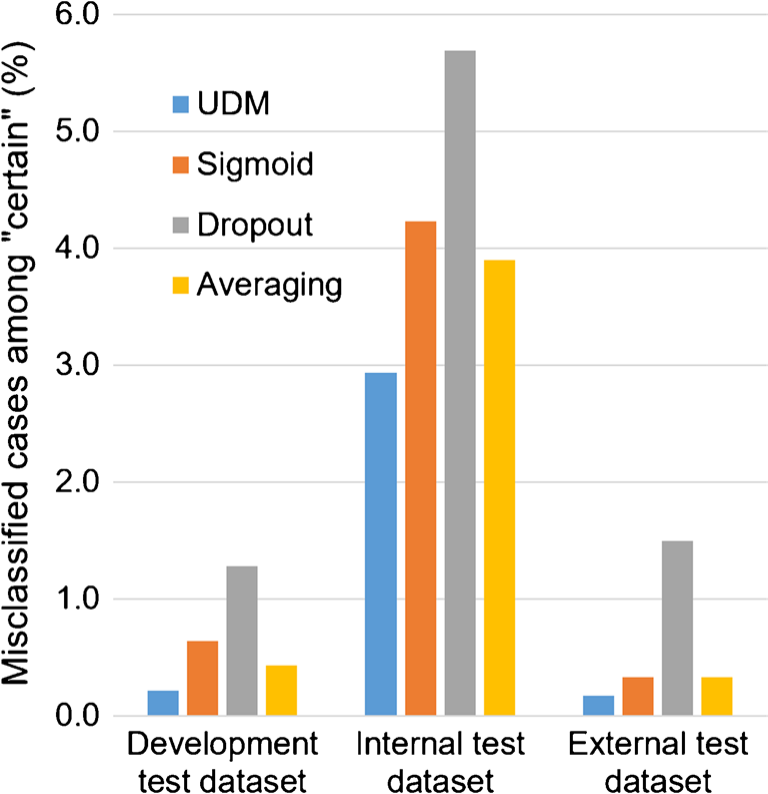
Uncertainty detection performance: proportion of misclassified cases among “certain” cases. For the three comparison methods (“sigmoid”, “dropout”, “model averaging”) the proportion of “uncertain” cases in the whole testset was fixed to be the same as for the UDM, separately for each testset (4.3%, 3.9%, and 6.5% for the test sample from the development dataset, the internal test dataset, and for the external test dataset, respectively)

The proportion of misclassified cases among “certain” cases is shown in Fig. 5.

In the test sample from the development dataset, 42.9% of the cases identified as “uncertain” by the UDM were misclassified by the NEfC, the remaining 57.1% “uncertain” cases were correctly classified.

## Discussion

The primary finding of this study was that the novel UDM identified a large proportion of the DAT-SPECT that were misclassified by the NEfC (Fig. 4a). It outperformed all tested comparison methods in this task, independent of the test dataset. The application scenario in clinical routine is as follows: both, NEfC and UDM, are applied to the DAT-SPECT to be evaluated. If the UDM identifies the DAT-SPECT as “uncertain”, careful visual inspection of the images by an experienced reader might result in overruling the NEfC decision. In particular, the experienced reader might assess the DAT-SPECT as inconclusive (and might recommend repeat or follow-up DAT-SPECT [53]). We hypothesize that in this way the UDM can contribute to improved diagnostic accuracy of DAT-SPECT for the etiological diagnosis of parkinsonism. This should be tested in future prospective studies. If the UDM identifies the DAT-SPECT as “certain”, the automatic classification by the NEfC is very reliable. In the independent test sample from the development dataset as well as in the external test sample from the PPMI, the proportion of misclassified “certain” cases was only about 0.2%, that is, lower than intra-reader variability of the visual interpretation of DAT-SPECT [26, 39].

In the test set from the development dataset, nine of ten misclassified cases were flagged as “uncertain” by the UDM, indicating 90% sensitivity of the UDM to identify misclassified cases. Retrospective visual inspection of the single misclassified case (10%) that was not flagged as “uncertain” by the UDM revealed that the gold standard label might be questioned in this case (Supplementary Fig. 1). This suggests that limitations of the gold standard label might have resulted in some underestimation of UDM performance.

Concerning clinical utility, only about 5% of all test cases were flagged as “uncertain” by the UDM (about 2.5% of the correctly classified cases). Thus, clinical utility of the UDM is not limited by an overly large proportion of “uncertain” cases. Furthermore, 5% “uncertain” cases according to the UDM is in line with 5–10% visually inconclusive cases among CUPS in clinical routine [21, 22].

In the test sample from the development dataset, about half of the “uncertain” cases were correctly classified by the NEfC. This is not a limitation of the UDM, but it is required for consistency, assuming that about 50% of the borderline cases are correctly classified by the NEfC (more or less by chance). Thus, the proportion of about 50% correctly classified “uncertain” cases suggests that most of these cases were rightly identified as “uncertain”. This was confirmed by retrospective visual inspection (Supplementary Fig. 1).

The UDM achieved about the same performance in the external test dataset from the PPMI than in the test sample from the internal development dataset (92.3 versus 90.0% sensitivity for labeling misclassified cases as “uncertain”), despite the fact that it was trained in the internal development dataset only and that the image characteristics were notably different between both datasets (lower striatum-to-background contrast in the external test dataset compared to the development dataset, Fig. 1). This demonstrates robustness of the UDM with respect to reasonable variability of image characteristics typically encountered in practice. This is required for widespread clinical use without the need for strict harmonization of acquisition and reconstruction protocols between cameras and sites. Harmonization is feasible in prospective clinical studies, but it is difficult in clinical routine. Even in the internal test dataset acquired with multiple-pinhole collimators (providing about 50% higher striatum-to-background contrast compared to the DAT-SPECT in the training dataset, Fig. 1), the UDM flagged 52.6% of the misclassified cases as “uncertain” and, therefore, demonstrated useful for improvement of classification accuracy also in this exceptional dataset.

The large between-datasets variability regarding the image characteristics was intended in the current study in order to allow testing the between-site/between-camera robustness of the proposed UDM. But it should be noted that adjusting the image characteristics of new DAT-SPECT images to be classified to the image characteristics of the dataset used for the training of the NEfC and the UDM has the potential to further improve their performance (subsection “Impact of between-datasets harmonization” in the Supplementary Material).

Uncertainty detection by the UDM is not based on a threshold, in contrast to the comparison methods that use a threshold on the (mean) sigmoid output of the CNN. This is an advantage of the UDM, because careful calibration of a threshold can be difficult on unseen data (as indicated by the large variability of the threshold parameter t on the sigmoid output between the different comparison methods and between the different test datasets, Supplementary Table 3).

It might be noted that the proposed UDM approach for the detection of “uncertain” cases, based on the combination of a highly sensitive and a highly specific classificator, is not restricted to CNN-based binary classification of DAT-SPECT. We hypothesize that this approach is also useful for other binary classification tasks and other classification methods (e.g., support vector machines).

Concerning possible reasons for borderline findings in DAT-SPECT, first, structural/vascular lesions should be ruled out by combined reading of DAT-SPECT with structural imaging, preferably MRI, as recommended by prodecure guidelines for DAT-SPECT [54]. When doing so, it should be taken into account that the striatal DAT-SPECT signal can be affected not only by lesions in the striatum or in the substantia nigra, but also by subcortical white matter lesions [55]. Next, it should be noted that motor symptoms typically manifest in Parkinson’s disease only after (unilateral) putaminal DAT loss has reached about 50% [56]. Thus, borderline DAT-SPECT is not typical of Parkinson’s disease in the motor phase. This does not rule out borderline DAT-SPECT findings in the premotor phase of Parkinson’s disease, for example in patients referred to DAT-SPECT because of idiopathic rapid eye movement sleep behavior disorder that can precede motor symptoms in α-synucleinopathies including Parkinson’s disease and multiple system atrophy. It also does not rule out borderline DAT-SPECT findings in patients with dementia with Lewy bodies, which can present with rather uniform signal reduction in the entire bilateral striatum, that is, without the caudate-to-putamen gradient that is characteristic for Parkinson’s disease [57]. A study on the utility of follow-up DAT-SPECT in case of inconclusive baseline DAT-SPECT found follow-up DAT-SPECT after 22 ± 14 months to show clearly normal striatal [^123^I]FP-CIT uptake in about 70% of the cases [53]. This suggests that the mild abnormality of the striatal signal in the baseline SPECT was an artifact, most likely caused by head motion during the SPECT acquisition [53]. However, about 20% of the patients showed clear progression of the baseline abnormality at the follow-up SPECT, suggesting nigrostrial degeneration that at baseline was at an too early stage to be clearly identified in the DAT-SPECT [53]. The vast majority of patients with clear progression were older than 60 years [53]. Thus, nigrostriatal degeneration might not be ruled out in case of borderline DAT-SPECT, particularly in patients older than 60 years, but this is more of an exception. The final interpretation after the follow-up SPECT in this previous study (as either normal or indicative of nigrostriatal degeneration) did not depend on the time interval between baseline and follow-up SPECT. As a consequence, the authors recommended a rather short delay of 6–12 months for the follow-up SPECT in case of inconclusive baseline findings [53]. Drug interactions, too, can complicate the interpretation of DAT-SPECT [58].

Clinical information can be useful to support the interpretation of borderline DAT-SPECT. For example, the reduction of the putaminal DAT-SPECT signal is usually more pronounced in the brain hemisphere contralateral to the side of the body that is more strongly affected by the motor symptoms [59, 60]. Thus, some minor left–right asymmetry of the DAT-SPECT signal in the posterior putamen to the disadvantage of the hemisphere ipsilateral to the more strongly affected side of the body is not very likely due to nigrostriatal degeneration.

Limitations of the current study include the following. First, the UDM based on the combination of two classificators is restricted to the binary discrimination between “uncertain” and “certain” cases, it does not provide a (more or less continuous) certainty or probability estimate (e.g., in %). The latter might be achieved by combining multiple classificators covering the whole range from very high sensitivity to very high specificity. However, the added value from a continuous certainty measure in clinical routine beyond the binary discrimination between “uncertain” and “certain” cases is not clear (it requires cut-offs to derive specific recommendations), particularly for applications in which the classification accuracy is rather high from the beginning (as in DAT-SPECT). Second, no attempts were made to increase robustness with respect to variability of image characteristics between DAT-SPECT from different cameras and/or sites. We hypothesize that the robustness of both, NEfC and UDM, with respect to variability of the image characteristics can be increased by using heterogeneous datasets for the training [18]. This should be tested in future studies. Finally, two-dimensional slab views were used as input to both, the NEfC and the UDM, because pilot experiments had not demonstrated an added value of the full three-dimensional images compared to the two-dimensional slab views regarding CNN-based clasification accuracy. However, an added value of the full three-dimensional DAT-SPECT images regarding the identification of uncertain cases by the UDM cannot be ruled out. This also should be tested in future studies.

In conclusion, the proposed uncertainty detection module provides reliable identification of borderline [^123^I]FP-CIT SPECT with high probability of misclassification. It is rather robust against reasonable between-sites variability of the image characteristics and, therefore, does not require strict harmonization of the image characteristics. We expect that combining CNN-based classification with the uncertainty detection module will improve the utility and the acceptance of automatic interpretation of [^123^I]FP-CIT SPECT for widespread use in clinical routine. The proposed UDM approach (“high sensitivity versus high specificity”) might be useful also for DAT imaging with other ligands and for other binary classification tasks.

### Supplementary Information

Below is the link to the electronic supplementary material.

Supplementary file1 (DOCX 266 KB)

## Data Availability

The inference code and the trained network weights are publicly available at https://github.com/ThomasBudd/dat_spect_ud.

## Abbreviations

CNN: Convolutional neural network
CUPS: Clinically uncertain parkinsonian syndrome
DAT-SPECT: Dopamine transporter single photon emission computed tomography
DVR: Distribution volume ratio
NE: Network ensemble
NEfC: Network ensemble for classification (with high overall accuracy)
PPMI: Parkinson’s Progression Markers Initiative
UDM: Uncertainty detection module
