# Supplementary material for: Automated identification of uncertain cases in deep learning-based classification of dopamine transporter SPECT to improve clinical utility and acceptance
Source: Eur J Nucl Med Mol Imaging. 2023 Dec 22;51(5):1333–44. doi: 10.1007/s00259-023-06566-w (PMC10957699; doi:10.1007/s00259-023-06566-w)
Supplement: Supplementary file 1 — Supplementary file1 (DOCX 266 KB) [file 259_2023_6566_MOESM1_ESM.docx]

**Supplementary Information**

**Automated identification of uncertain cases in deep learning-based classification of dopamine transporter SPECT to improve clinical utility and acceptance**

Thomas Budenkotte^1^, Ivayla Apostolova^1^, Roland Opfer^2^, Julia Krüger^2^, Susanne Klutmann^1^, Ralph Buchert^1^

^1^Department of Diagnostic and Interventional Radiology and Nuclear Medicine, University Medical Center Hamburg-Eppendorf, Hamburg, Germany

^2^jung diagnostics GmbH, Hamburg, Germany

**Corresponding author:** Ralph Buchert, Department of Diagnostic and Interventional Radiology and Nuclear Medicine, University Medical Center Hamburg-Eppendorf, Martinistr. 52, 20246 Hamburg, Germany, Email: [r.buchert@uke.de](mailto:r.buchert@uke.de), Phone: +49 (0)40 7410-54347, Fax: +49 (0)40 7410-40265, ORCID ID 0000-0002-0945-0724

**Acknowledgements:** Data used in the preparation of this article were obtained from the Parkinson’s Progression Markers Initiative (PPMI) database (www.ppmi-info.org/access-data-specimens/download-data). For up-to-date information on the study, visit [www.ppmi-info.org](http://www.ppmi-info.org). PPMI – a public-private partnership – is funded by the Michael J. Fox Foundation for Parkinson’s Research and funding partners. For up-to-date information about all of the PPMI funding partners visit www.ppmi-info.org/about-ppmi/who-we-are/study-sponsors.

***Impact of between-datasets harmonization***

The high-resolution 2-dimensional slab view images of the internal test dataset (Figure 1 in the manuscript) were smoothed to about the same spatial resolution as the 2-dimensional slab view images of the development dataset (by filtering with an isotropic Gaussian kernel of 9 mm full-width-at-half-maximum). Automatic classification of the smoothed slabs by the network ensemble for classification (NEfC, trained in the training subset of the development dataset) demonstrated improved overall accuracy (from 0.941 to 0.953) compared to automatic classification of the original (unsmoothed) slabs in the internal test dataset. The improvement was mainly driven by improved sensitivity (from 0.887 to 0.911). These findings demonstrate that the accuracy of automatic CNN-based classification of DAT-SPECT can be improved by adjusting the image characteristics of the cases to be classified to the image characteristics of the dataset used for the CNN’s training. The proportion of cases in the internal test dataset labelled as uncertain by the UDM was not changed by the smoothing. This finding further supports the robustness of the proposed UDM with respect to variable image characteristics.

**Supplementary Tab. 1** Hyper-parameters of the intensity-based augmentations. U[x,y] denotes a uniform distribution with minimum x and maximum y

| Operation | Application probability | Magnitude |
| --- | --- | --- |
| Additive Gaussian noise | 0.15 | U[0, 0.1] |
| Gaussian blurring | 0.1 | U[0.5, 1.5] |
| Brightness | 0.15 | U[0.7, 1.3] |
| Contrast | 0.15 | U[0.65, 1.5] |
| Gamma | 0.15 | U[0.7, 1.5] |

**Supplementary Tab. 2** Classification performance of the „high sensitivity“ network ensemble and of the „high specificity“ network ensemble of the UDM

|  | „High sensitivity“ | | „High specificity“ | |
| --- | --- | --- | --- | --- |
| Dataset | Sensitivity | Specificity | Sensitivity | Specificity |
| Test sample from the development dataset | 1.000 | 0.958 | 0.952 | 0.996 |
| Internal test dataset | 0.957 | 0.997 | 0.884 | 1.000 |
| External test dataset | 1.000 | 0.816 | 0.989 | 0.995 |

**Supplementary Tab. 3** Thresholds t on the (mean) sigmoid output for identification of „uncertain“ cases with the comparison methods (a scan was flagged „uncertain“ if the mean sigmoid output was in the interval [0.5-t, 0.5+t]). The thresholds were fixed such that the number of „uncertain“ scans was the same as with the UDM

|  | Sigmoid | Dropout | Model averaging |
| --- | --- | --- | --- |
| Test sample from the development dataset | 0.06 | 0.43 | 0.07 |
| Internal test dataset | 0.28 | 0.47 | 0.25 |
| External test dataset | 0.01 | 0.45 | 0.02 |

**
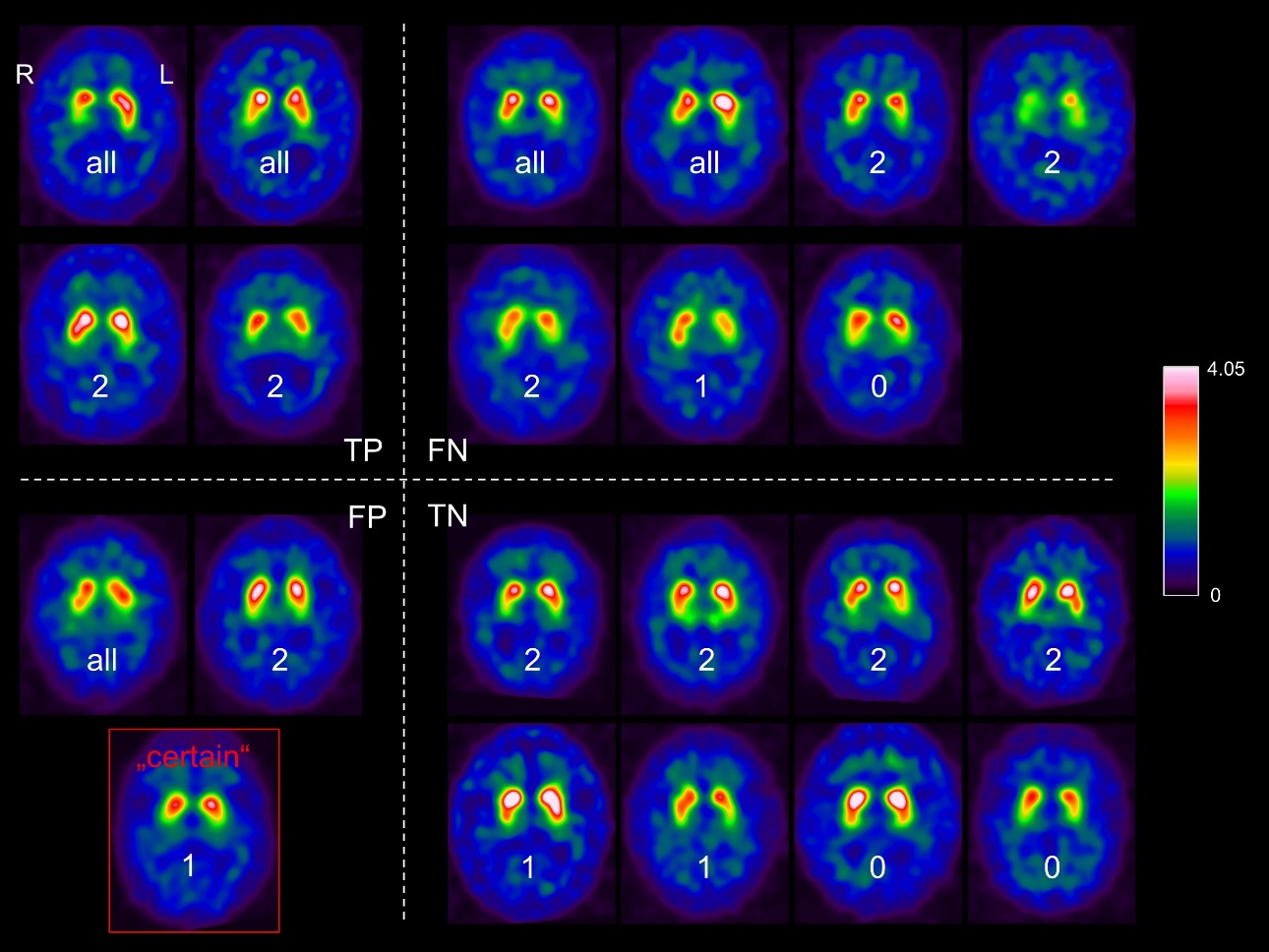
**

**Supplementary Fig. 1** Two-dimensional 12 mm slabs of all 21 scans in the test subset from the development dataset that were flagged as „uncertain“ by the UDM. The scans are arranged according to the automatic classification by the NEfC: true positives (TP), false negatives (FN), false positives (FP) and true negatives (TN). The number of comparison methods that also flagged the scan as „uncertain“ is given for each scan (number below the striata). The single case that was misclassified by the NEfC but not flagged as „uncertain“ by the UDM is shown on the bottom left (red outline). The case was classified „normal“ by 2 readers, it was classified „reduced“ by the remaining reader. Thus, the gold standard label (majority vote) was „normal“. The NEfC classified the case as „reduced“, that is, FP with reference to the gold standard
